# An Account of a Rupture of the Uterus during Labour

**Published:** 1813-10

**Authors:** 


*For the Medical and Physical Journal.*

### *An* ACCOUNT *of a* RUPTURE *of the* UTERUS *during* LABOUR.

ON Thursday the 29th of July last, I was requested to inquire into the cause of death in a poor woman who had died in labour, undelivered, the day preceding. Upon calling at the house of the deceased with the view of making the necessary arrangements, I found the body in a high state of putrefaction, though life had been extinct little more than twenty-four hours. The belly was distended to an astonishing size; the thighs and legs were emphysematous and œdematous; vesications containing a colored fluid had arisen in various parts of the body; the face was swelled, and had become quite black: in short, the dead body exhibited the most disgusting spectacle I ever beheld.

In the course of that evening, in the presence of two other medical gentlemen, I examined the body. On making a puncture into the abdominal cavity, a considerable quantity of a most offensive gas escaped, which not only affected the air in the room in a most nauseous manner, but even that of the whole house. Upon dividing the abdominal parietes in the course of the linea alba, the fœtus presented itself to view, lying in the cavity of the abdomen, completely out of the uterus, across the brim of the pelvis, with the left side of its head upon the right ilium, with its breech and left thigh upon the left ilium, having its back inclined towards the pubes, and its belly towards the lower part of the spine. On raising the fœtus, the placenta was seen lying upon the anterior peritoneal surface of the uterus, which, as well as the child, was was completely expelled out of its cavity; in the pelvis, there was but a small quantity of bloody fluid. The fœtus was large in size, and showed evident marks of commencing putrefaction. The uterus was tolerably well contracted ; as much so, in the opinion of the gentlemen present, as that organ usually is found a few hours after delivery. On making a further inquiry, a large laceration, the extent of the whole anterior diameter of the uterus, was observed about the middle of its body: through this opening, in the enlarged state of the uterus, the child and placenta had passed. The intestinal canal was inflated through its whole extent. The other viscera of the abdomen, as far as they were examined, assumed a healthy appearance.

From the attending midwife, and the patient's friends, I obtained some particulars of the progress of the labour, and other circumstances. This poor woman, æt. 35, was of a corpulent habit, the tendency to which had very considerably increased during her last pregnancy. She had born several children, and usually had lingering labours. She began to be in labour on the Monday evening preceding, and early on the Tuesday morning, the midwife was sent for, who arrived about five o'clock. Before the arrival of the midwife, according to the representation of her friends, she was in strong labour. On examination, the midwife could not determine the presenting part of the child ; the os uteri was a little open, and the membranes entire. The pains continued to increase ; and after some hours the membranes either gave way spontaneously, or were ruptured. After this, the midwife felt some part of the child very high, which she believed to be the head, and waited patiently in expectation of its descent. As this poor woman had, in her former labours, suffered considerably, the midwife's apprehensions were not excited by the child's not advancing. About noon on Tuesday, the patient complained to her sister, after a violent labour-pain, that *her burden* had suddenly risen much higher ; that she had a pain about her heart, (as she said) with very considerable difficulty of breathing, and a sense of suffocation. From this time, the regular pains almost ceased ; but the poor woman complained of great uneasiness in the belly with the sense of suffocation ; yet she was not entirely prevented from moving about. Shortly afterwards she was seized with vomitings of a black offensive substance, which continued at intervals during the remainder of life.

Thus hour after hour passed away till the next morning, the midwife being in constant attendance, and occasionally making an examination, but found no advance in the labour. Early on the Wednesday morning, the belly was observed to be be swelled more than usual, and after a while symptoms of a convulsive nature made their appearance; upon which, the midwife requested the assistance of an eminent practitioner, but before he was able to see the patient, she had breathed her last about noon on Wednesday.

In this case, rupture of the uterus occurred from the contractions of that organ upon the child in an adverse position; and the opening was sufficiently extensive to allow of the sudden passage of the child and placenta into the cavity of the abdomen, after which the uterus contracts itself. Under this unfortunate accident, it rarely happens that the child is entirely protruded out of the uterine cavity: it commonly is found partly in the uterus, partly in the abdomen. It also seldom happens that a patient lives so long after the accident as in the present instance: her continuance under her sufferings might possibly be owing to the perfect escape of the child from the uterus, and to its subsequent contraction. I enquired particularly respecting any appearance of external hemorrhage, and was answered that there was very little colored discharge, but that little was very offensive. The contracted state of the uterus explains a want of hemorrhage. In this instance, after the accident had taken place, and the uterus had contracted itself, delivery by the natural passages would have been impossible; the patient must have been left to her fate.

I will make no comments on the practice a judicious operator would have pursued had he been called before the rupture of the membranes: the very nature of the presentation would have determined that point. Difference of opinion has existed in the minds of medical men as to the best practice under cases of rupture of the uterus: for my part, immediate delivery by turning the child, when that is practicable, seems the only alternative offering a chance either to the mother or child. In the above case, the accident was not suspected.

